# Causes of HIV Treatment Interruption during the Last 20 Years: A Multi-Cohort Real-Life Study

**DOI:** 10.3390/v15030720

**Published:** 2023-03-10

**Authors:** Andrea De Vito, Elena Ricci, Barbara Menzaghi, Giancarlo Orofino, Canio Vito Martinelli, Nicola Squillace, Lucia Taramasso, Giuseppe Vittorio De Socio, Chiara Molteni, Laura Valsecchi, Cecilia Costa, Benedetto Maurizio Celesia, Giustino Parruti, Giovanni Francesco Pellicanò, Eleonora Sarchi, Antonio Cascio, Giovanni Cenderello, Katia Falasca, Antonio Di Biagio, Paolo Bonfanti, Giordano Madeddu

**Affiliations:** 1 Unit of Infectious Diseases, Department of Medicine, Surgery and Pharmacy, University of Sassari, 07100 Sassari, Italy; giordano@uniss.it; 2 Fondazione ASIA Onlus, Buccinasco, 20090 Milan, Italy; 3 Unit of Infectious Diseases, ASST della Valle Olona, Busto Arsizio Hospital, 21052 Busto Arsizio, Italy; 4 Unit of Infectious Diseases, “Divisione A”, Amedeo di Savoia Hospital, 10149 Torino, Italy; 5 SOD Malattie Infettive e Tropicali, AOU Careggi, 50100 Firenze, Italy; 6 Infectious Diseases Clinic, Fondazione IRCCS San Gerardo dei Tintori, University of Milano-Bicocca, 20126 Monza, Italy; 7 Infectious Disease Clinic, IRCCS Policlinico San Martino Hospital, 16132 Genoa, Italy; 8 Clinic of Infectious Diseases, Department of Medicine, Azienda Ospedaliera di Perugia, Santa Maria Hospital, 06100 Perugia, Italy; 9 Infectious Disease Unit, Ospedale A. Manzoni, 23900 Lecco, Italy; 10 Infectious Disease Unit (I Division), ASST Fatebenefratelli Sacco, 20157 Milan, Italy; 11 Infectious Diseases Department, SOC 1, USLCENTROFIRENZE, Santa Maria Annunziata Hospital, 50012 Florence, Italy; 12 Unit of Infectious Diseases, University of Catania, ARNAS Garibaldi, 95124 Catania, Italy; 13 Infectious Diseases Unit, Pescara General Hospital, 66020 Pescara, Italy; 14 Unit of Infectious Diseases, Department of Human Pathology of the Adult and the Developmental Age “G. Barresi”, University of Messina, 98122 Messina, Italy; 15 Infectious Diseases Unit, SS. Antonio e Biagio e Cesare Arrigo Hospital, 15121 Alessandria, Italy; 16 Infectious and Tropical Diseases Unit, Department of Health Promotion, Mother and Child Care, Internal Medicine and Medical Specialties (PROMISE), University of Palermo, 90133 Palermo, Italy; 17 Infectious Disease Unit, Sanremo Hospital, 18038 Sanremo, Italy; 18 Clinic of Infectious Diseases, Department of Medicine and Science of Aging, University “G. d” Annunzio’ Chieti-Pescara, 66100 Chieti, Italy

**Keywords:** ART, antiretroviral treatment, safety, interruption, durability, INSTI, PI, NNRTI

## Abstract

In the last years, many antiretroviral drugs (ART) have been developed with increased efficacy. Nowadays, the main reasons for treatment switches are adverse events, proactive strategy or simplification. We conducted a retrospective cohort study to investigate the reason for treatment interruption in the last 20 years. We merged data of eight cohorts of the SCOLTA project: lopinavir/r (LPV), atazanavir/r (ATV), darunavir/r or /c (DRV), rilpivirine (RPV), raltegravir (RAL), elvitegravir/c (EVG), dolutegravir (DTG) and bictegravir (BIC). We included 4405 people with HIV (PWH). Overall, 664 (15.1%), 489 (11.1%), and 271 (6.2%) PWH interrupted the treatment in the first, second, and third years after starting a new ART. Looking at the interruption in the first year, the most frequent causes were adverse events (3.8%), loss to follow-up (3.7%), patients’ decisions (2.6%), treatment failure (1.7%), and simplification (1.3%). In the multivariate analysis regarding experienced patients, treatment with LPV, ATV, RPV or EVG/c, having less than 250 CD4 cells/mL, history of intravenous drug use, and HCV positivity were associated with an increased risk of interruption. In naive people, only LPV/r was associated with an increased risk of interruption, while RPV was associated with a lower risk. In conclusion, our data on more than 4400 PWH show that adverse events have represented the most frequent cause of treatment interruptions in the first year of ART (3.84%). Treatment discontinuations were more frequent during the first year of follow-up and decreased thereafter. First-generation PI in both naïve and experienced PWH, and EVG/c, in experienced PWH, were associated with a higher risk of treatment interruptions.

## 1. Introduction

Thanks to the introduction of antiretroviral regimens (ART), HIV infection has become a chronic condition in which, at present, therapy should be continued lifelong [1]. Furthermore, effective ART has an important impact on public health by preventing the transmission of HIV [2,3]. Several new treatments and strategies have been proposed in the last twenty years, with the introduction of second generation boosted PI, integrase inhibitors (INSTI), and second generation of NNRTI. The guidelines have undergone multiple changes for the treatment strategy and the first-line regimens. Until 2015, the decision to start ART was based on the CD4 number and viral load, with the suggestion to begin antiretroviral treatment only if the CD4 count was below 350 cells/mm^3^ and the viral load was higher than 100,000 copies/mL [4]. With the publications of the INSIGHT START Study Group trial [5,6], it was proven that starting antiretroviral therapy in people with HIV (PWH) with CD4+ count >500 cells/mm^3^ provided significant benefits over waiting after the CD4+ count had declined to 350 cells/mm^3^. Also, the first-line regimens have been changed in the last 20 years. For example, in 2003 EACS guidelines, treatment with three different Nucleoside Reverse Transcriptase Inhibitors (NRTI) was suggested, while we now know how this approach can lead to the emergence of mutations and viral load rebound [7,8].

Many first-line regimens have been downgraded to alternative regimens since other more effective and better-tolerated treatments have been approved [9,10,11,12,13,14,15]. In particular, all protease inhibitors (PI) (lopinavir/ritonavir [LPV/r], atazanavir/ritonavir [ATV/r], and darunavir ritonavir or cobicistat [DRV/r or /c]), are not recommended for naïve PWH; only DRV/ is suggested as alternative regimens, and in treatment switch [16].

In the last EACS guidelines, INSTIs represent the preferred class for first-line therapy and as an option for optimization in experienced PWH due to their high efficacy, genetic barrier, and tolerability [12,13,17,18,19,20,21,22]. However, in the last few years, the scientific community focused on this class’s possible role in weight gain, particularly dolutegravir (DTG) [11,21,23,24]. Regarding Non-Nucleoside Reverse Transcriptase Inhibitors (NNRTI), only rilpivirine (RPV) and doravirine (DOR) are recommended by EACS guidelines, thanks to their excellent efficacy and safety [14,25,26,27,28].

The SCOLTA project is a multicenter observational study started in 2002 following prospectively HIV-infected people who began to take newly introduced antiretroviral drugs. The project aims to identify toxicities and adverse effects (AEs) in a real-life setting. The SCOLTA project uses an online pharmacovigilance program and involves 29 Italian Infectious Disease Centers [29].

We aimed to investigate the reason for treatment interruption in the last 20 years during the first year and the entire follow-up, collecting data for all interruption causes, including adverse events.

## 2. Materials and Methods

We conducted a retrospective cohort study, merging the data of eight different cohorts of the SCOLTA project: LPV/r (2002–2006), ATV (2003–2008), DRV/r or /c (2006–2019), RPV (2013–2017), raltegravir (RAL; 2007–2014), elvitegravir/c (EVG; 2014–2019), dolutegravir (DTG; 2014-ongoing) and bictegravir (BIC; 2019-ongoing) cohorts. The cohorts’ complete data collection and follow-up procedures have been previously described [29].

Briefly, we collected demographical information, risk factors for HIV infection, viral-immunological data, and the causes of treatment interruption. Discontinuation was defined as PLW stopping the use of the cohort drug.

Both ART naïve- and experienced patients can be included in SCOLTA if they are >18 years old and sign a written informed consent. Clinical data collected include sex, age, ethnicity, weight, height, CDC stage, and previous ART history. Laboratory data include HIV-RNA, CD4+ cell count, CD4/CD8 ratio, and biochemical data. All information is prospectively collected in a central database every six months in an anonymized form.

All AEs causing drug discontinuation are collected when they occur and categorized according to a standardized toxicity grade scale [30]. Virological failure was defined at the time of the first of two consecutive HIV-RNA above the threshold of 50 copies/mL occurring in people who had previous HIV RNA < 50 copies/mL [29]. 

The original study protocol was approved on 18 September 2002, and new protocol amendments were approved on 13 June 2013, and 3 March 2020, by the coordinating center at Hospital “L. Sacco”-University of Milan and after that by all participating centers. In addition, written consent for study participation was obtained from all participants. The study was conducted in accordance with the ethical standards laid down in the 1964 Declaration of Helsinki and its later amendments and by Italian national laws.

### Statistical Analysis

Data were described using mean and standard deviation (SD) for normally distributed continuous variables, median and interquartile range (IQR) for not normally distributed continuous variables, and frequency (%) for categorical and ordinal variables. Rates were calculated as the number of discontinuations per 100 patient-years (PY).

Using unconditional multiple logistic regression, we estimated rate ratios (RR) and corresponding 95% confidence interval (CI) of regimen discontinuation for any causes over the first year of treatment. In the logistic regression equation, we included the variables significantly associated with the outcomes after accounting for age and sex. The Cox proportional-hazards model was used to estimate the hazard ratio (HR) and 95% CI of regimen discontinuation for any cause over the follow-up period. DTG cohort was used as the reference in both analyses. 

The significance level was set at <0.05. Statistical analysis was performed using the SAS/STAT statistical package (version 9.4; SAS Institute Inc., Cary, NC, USA).

## 3. Results

We included 4405 people, 690 treated with LPV/r, 527 with ATV/r, 646 with DRV/r or DRV/c, 344 with RPV, 468 with RAL, 332 with EVG/c, 1126 with DTG, and 272 with BIC. The characteristics of PWH by regimen are reported in Table 1.

Overall, 1424 (32.32%) PWH interrupted the treatment; in particular, 664 (15.1%), 489 (11.1%), and 271 (6.2%) were interrupted in the first, second, and third year, respectively. Looking at the interruption in the first year, the most frequent causes were AEs (3.8%), loss to follow-up (3.7%), patients’ decisions (2.6%), treatment failure (1.7%), and simplification (1.3%). In particular, interruption due to AEs was higher in DTG, RPV, and LPV/r groups, while therapeutic failure was experienced more frequently in EVG/c, LPV/r, and ATV/r groups. In addition, there was a substantial loss to follow-up for LPV/r and ATV/r groups. Finally, the patient’s decision to interrupt the treatment proportion was higher for all boosted drugs (LPV, ATV, DRV, and EVG). The causes of interruption during the first year of treatment are summarized in Table 2.

Over the whole observation period, 1718 ART-experienced PWH interrupted the cohort regimens (discontinuation rate 14.8/100 py, 95% CI 14.0–15.6), whereas the corresponding figure was 17.8 (95% CI 15.7–19.6) for ART-naïve people (*p* = 0.007). Discontinuations due to adverse events had a rate of 2.5/100 PY (95% CI 2.5–2.9) and 4.0 (95% CI 3.2–5.1), respectively (*p* = 0.0001).

To identify the risk factors associated with all causes of regimen interruption during the first year of observation, we performed a multivariate analysis separately for ART-experienced and ART-naïve people. Available variables were analyzed as associated with interruption in a model adjusted for sex and age. The complete model included the variable significantly associated with regimen interruption at the age and sex-adjusted analysis (Table 3). In ART-experienced PWH, interruptions were considerably higher in association with female sex, age > 50 years, detectable HIV-RNA at study entry, CDC stage C and CD4+ > 500 cells/mm^3^. Compared to DTG, treatment with LPV, ATV, RPV or EVG/c were associated with an increased risk of interruption. 

In naïve people, the multivariate model showed that a higher risk of discontinuation was associated with LPV regimens. In contrast, ART-naïve PWH on RPV had a lower risk of interruption, as compared to DTG (Table 4).

During the first year, 133 interruptions due to adverse events occurred in ART-experienced and 36 in ART-naïve PWH. In ART-experienced PWH, discontinuations were associated with age >50 years (RR 2.02, 95% CI 1.40–2.92) and being on treatment with DRV (RR 0.52, 95% CI 0.28–0.95) and RAL (RR 0.22, 95% CI 0.08–0.61) as compared to DTG. In ART-naïve people, no variables were significantly associated with discontinuations due to adverse events.

**Figure 1:**
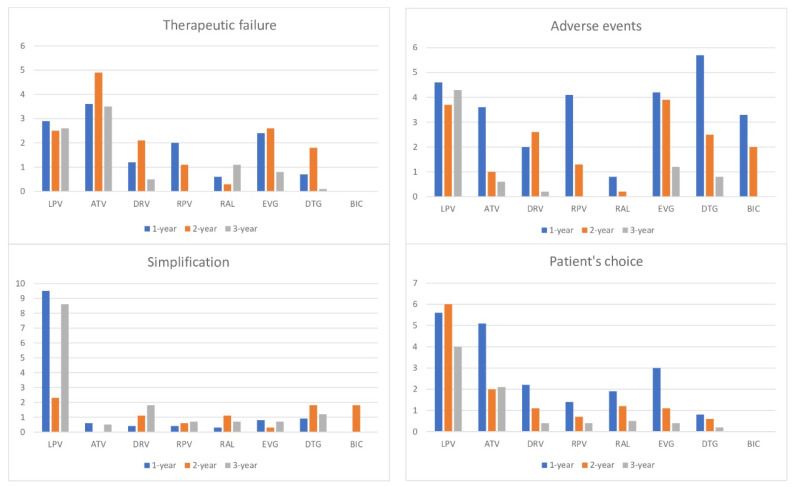
Percentage of discontinuation causes in patients enrolled in the SCOLTA project cohorts during the first, second and third year of treatment.

Looking at three years of follow-up, the proportion of interruptions due to adverse events decreased from 84/489 (17.2%) in the second to 28/271 (10.3%) in the third year (Figure 1).

Finally, we analyzed the discontinuations over the entire follow-up. As in the previous model, available variables were analyzed in a model adjusted for sex and age. In addition, the complete model included the variable significantly associated with regimen interruption at the age and sex-adjusted analysis.

At the multivariate analysis for all causes of interruption during the entire follow-up (Table 5), the group of ART-experienced PWH women, non-Caucasian people, PWID, and those who entered the cohorts with detectable HIV-RNA showed an increased risk of regimen discontinuation. A higher level of CD4+ cells at study entry was associated with a lower risk of interruption. Compared to DTG, a higher risk of discontinuation was observed in LPV, ATV, RPV, and EVG-treated PWH.

In the group of naïve PWH, HCV antibody positivity and treatment with LPV and RAL were associated with an increased risk of treatment interruption. On the contrary, starting therapy with RPV was associated with a lower risk (Table 6).

## 4. Discussion

In this study, we evaluated with the same methodology all discontinuations in PWH receiving different new antiretroviral drugs during 20 years of follow-up. We found a high discontinuation rate in the LPV/r cohort and a halving of discontinuation rates for the newer regimens. In naïve patients, the rates were comparable to other studies in the literature.

Toxicities have represented the leading cause of treatment interruption since the early 2000s. d’Arminio Monforte et al. in 2000 found that naïve PWH who did not experience toxicities discontinued the treatment in less than 10% of cases during the first year of therapy [31], while the treatment interruption due to AEs was 21.1%. In the Swiss cohort, a similar incidence of treatment interruption for toxicity between 2000–2005 was found [32]. Also, Robinson et al., in their cohort, found a percentage of interruption around 55%, in particular, 24.8% due to toxicity. In the multivariate analysis, IDU history and PI-based regimens were associated with an increased risk of discontinuation during the first year of treatment [33]. In 2010, Cicconi et al. analyzed the discontinuation between 1997 and 2007 in the ICONA cohort. Overall, they found that people who started treatment between 2003–2007 had a lower risk of discontinuation for toxicities but a higher for simplification [34]. In 2013, Abgrall et al. published a paper on 21,801 patients from 18 cohorts in Europe and North America who started first-line regimens between 2002 and 2009 [35]. The cumulative percentages of modification, interruption, and death during the first three years were 47, 12, and 2%, respectively. The main reason for discontinuation was AEs (40%). Patients on lopinavir/r and other protease inhibitors had higher rates of modification and interruption. In 2016 Kanters et al. conducted a network meta-analysis on all clinical trials published before 5 July 2015, finding that treatment with DTG and RAL was associated with lower discontinuation for AEs [15].

In naïve, the higher rates of discontinuations of LPV and RAL and in PWID and HCV antibody-positive PWH could represent an adherence proxy since the two antiretroviral drug regimens were primarily prescribed twice a day. The need to treat hepatitis C could partly explain the LPV discontinuation in the HCV subgroups due to pharmacokinetic interactions. Also, RAL discontinuation may be partly linked to simplification requests by PWH.

Another interesting paper by Di Biagio et al. found that between 2008 and 2014, the leading cause of discontinuation was not toxicity but simplification, thus confirming that the new drugs had a better safety profile [36].

If for naïve patients, many studies analyzed the incidence and predictor of discontinuation, for experienced patients, there are fewer studies, and many explored the discontinuation only for the second-line regimens. Moreover, most studies are conducted in resource-limited countries [37,38,39]. If it is true that EACS 11.0 guidelines removed first-generation PIs from the recommended and alternative treatment, these drugs represent the preferred second-line choice in many countries. Ross et al. conducted a multicenter cohort study between 2015 and 2017 in 12 Asia-Pacific sites about second-line treatment; among 1378 PWH, 93% of people received regimens with PIs (55% LPV, 39% ATV); of these patients, 7% interrupted the treatment due to virologic failure [40]. Onoya et al. conducted in 2016 a retrospective study to analyze predictors of early drug substitutions and treatment interruptions in a multicenter South African cohort between 2004 and 2013. Overall, 11.7% had a drug substitution, and 6.3% interrupted the treatment. All patients in their cohort had a PIs-based treatment containing LPV/r or ATV/r [38]. Also, most of the second-line treatment in Brazil was PI-based (91%) [39]. Regarding INSTI-based regimens, De La Mata et al. analyzed 598 treatment-experienced PWH, of which 199 started second-line treatment containing INSTI, and 399 patients were considered highly experienced [41]. Among the second-line treatment group, 5.4% discontinued the treatment. The only factor associated with an increased risk of discontinuation in their cohort was female gender (HR 2.53 (95% CI: 1.10–5.82); female gender was associated with an increased risk of discontinuation also in our study, independently from the type of treatment (RR 1.18, 95%CI 1.02–1.37). In our cohort, there was an high interruption of DTG in the first year for adverse events, in part due to SNC adverse events, already described in many studies [17,42,43,44]. However, DTG interruptions due to AEs were counterbalanced by lower proportion of discontinuations due to other causes and rapidly declined over the study period, thus resulting in the RRs and HRs showed in Table 3, Table 4, Table 5 and Table 6. The comparatively low number of discontinuations for each cause, the necessity for competing risk analyses, and adjustment for repeated analyses would have resulted in non-statistically significant findings, with risk estimates and low clinical interest confidence interval.

Our study has some limitations. Firstly, it is a retrospective study, and the data were from an existing database. Secondly, there is a lack of information such as stratification of the number of MSM and heterosexual transmissions, clinical information about patients’ comorbidities and data about the treatment adherence. Furthermore, not all drug regimens are present in our study; for example, the EFV cohort is not present. However, many drugs no longer recommended in Europe represent the first choice in many resource-limited countries, in particular, efavirenz (EFV) as first-line regimens, and LPV or ATV as savage treatment, in combination with two NRTI. The strengths of this study are the real-life setting, the large sample size, the inclusion of many regimens, and the extensive period considered.

## 5. Conclusions

Our data on more than 4400 PWH show that adverse events have represented the most frequent cause of treatment interruptions. Treatment discontinuations were more frequent during the first year of follow-up and decreased thereafter. First-generation PI in both naïve and experienced PWH, and EVG/c, in experienced PWH, were associated with a higher risk of treatment interruptions.

## Figures and Tables

**Table 1:** Characteristics of 4405 people living with HIV, by SCOLTA cohort.

	LPV		ATV		DRV		RPV		RAL		EVG		DTG		BIC		*p*-Value *
	N = 690 15.7%		N = 527 12.0%		N = 646 14.7%		N = 344 7.8%		N = 469 10.6%		N = 332 7.5%		N = 1125 25.5%		N = 242 6.2%		
Period	2002–2006		2003–2008		2006–2019		2013–2017		2007–2014		2014–2019		2014–Ongoing		2019–Ongoing		
**Male gender**	502	72.8	357	67.7	475	73.5	250	72.7	314	67.0	255	76.8	842	74.8	217	79.8	0.0003
**Age,** years (mean ± SD)	40.3 ± 7.8		42.8 ± 8.1		45.8 ± 9.3		43.6 ± 10.2		45.8 ± 9.2		43.8 ± 10.8		48.1 ± 12.0		49.1 ± 11.8		<0.0001
Caucasian ethnicity	633	91.7	493	93.5	607	94.0	316	91.9	438	93.4	301	90.7	1024	91.0	243	89.3	0.12
**Risk factor for HIV acquisition**																	
Heterosexual	236	34.2	189	35.9	195	30.2	153	44.5	163	34.8	126	38.0	417	37.1	105	38.6	
MSM	139	20.1	81	15.4	132	20.4	118	34.3	97	20.7	121	36.4	376	33.4	83	30.5	
PWID	268	38.8	223	42.3	228	35.3	54	15.7	168	35.8	53	16.0	199	17.7	47	17.3	
Other/unknown	47	6.8	34	6.4	91	14.1	19	5.5	41	8.7	32	9.6	133	11.8	37	13.6	<0.0001
**HbsAg positive**	50	7.2	27	5.1	40	6.2	20	5.8	28	6.0	31	9.3	31	2.8	26	9.6	<0.0001
**HCV positive**	267	38.7	221	41.9	238	36.8	57	16.6	172	36.7	65	19.6	240	21.3	58	21.3	<0.0001
**CDC stage**																	
A	231	33.5	169	32.1	170	26.3	222	64.5	142	30.3	170	51.2	571	50.8	158	58.1	
B	187	27.1	170	32.3	242	37.5	76	22.1	143	30.5	88	26.5	305	27.1	61	22.4	
C	272	39.4	188	35.7	234	36.2	46	13.4	184	39.2	74	22.3	249	22.1	53	19.5	<0.0001
**Naïve**	135	19.6	18	3.4	47	7.3	103	29.9	27	5.8	100	30.1	283	25.2	36	13.2	<0.0001
**Detectable HIV-RNA** (experienced, *n* = 3656)	488	87.9	347	68.2	335	55.9	36	14.9	253	57.2	61	26.3	142	16.7	20	8.5	<0.0001
**CD4 cells/mm^3^**																	
<250	385	55.8	190	36.1	249	38.5	31	9.0	169	36.0	83	25.0	201	17.9	23	8.5	
250–500	220	31.9	219	41.6	196	30.3	122	35.5	172	36.7	92	27.7	278	24.7	67	24.6	
>500	84	12.2	118	22.4	196	30.3	191	55.5	126	26.9	157	47.3	623	55.4	182	66.9	<0.0001
**Year of first ART**																	
<1996	190	27.5	168	31.9	183	28.3	19	5.5	136	29.0	26	7.8	87	7.7	19	7.0	
1996–2002	411	59.6	265	50.3	232	35.9	61	17.7	207	44.1	58	17.5	233	20.7	41	15.1	
2003–2012	89	12.9	94	17.8	182	28.2	132	38.4	126	26.9	98	29.5	341	30.3	64	23.5	
>2012	.	.	.	.	49	7.6	132	38.4	.	.	150	45.2	464	41.2	148	54.4	<0.0001

* univariate analysis: means were compared using the analysis of variance, proportion using the chi-square or the Mantel-Haenszel test, as appropriate.

**Table 2:** Reason of interruption during the first year of treatment in 4405 people living with HIV, by SCOLTA cohort.

	LPV		ATV		DRV		RPV		RAL		EVG		DTG		BIC		Total	
	N = 690 15.7%		N = 527 12.0%		N = 646 14.7%		N = 344 7.8%		N = 469 10.6%		N = 332 7.5%		N = 1125 25.5%		N = 242 6.2%		N = 4405	
Overall	177	25.6	122	23.2	79	12.2	42	12.2	46	9.8	50	15.1	125	11.1	23	9.5	664	15.1
Adverse events	32	4.6	19	23.2	13	2.0	14	4.1	4	0.8	14	4.2	64	5.7	9	3.3	169	3.8
Clinical event *	6	0.9	1	0.2	0	0.0	1	0.3	2	0.4	0	0.0	0	0.0	0	0.0	10	0.2
Therapeutic failure	20	2.9	19	3.6	8	1.2	7	2.0	3	0.6	8	2.4	8	0.7	0	0.0	73	1.7
Pregnancy	0	0.0	1	0.2	0	0.0	0	0.0	0	0.0	0	0.0	1	0.1	0	0.0	2	0.1
Drug-Drug interactions	0	0.0	0	0.0	4	0.6	4	1.2	0	0.0	4	1.2	1	0.1	1	0.4	14	0.3
Death	4	0.6	4	0.8	5	0.8	1	0.3	3	0.6	2	0.6	6	0.5	1	0.4	26	0.6
Unknown	2	0.3	2	0.4	0	0.0	0	0.0	2	0.4	0	0.0	1	0.1	0	0.0	7	0.2
Optimization	4	0.6	1	0.2	1	0.2	0	0.0	0	0.0	0	0.0	2	0.2	0	0.0	8	0.2
Lost to follow-up	49	7.1	43	8.2	24	3.7	7	2.0	16	3.4	9	2.7	11	1.0	5	1.8	164	3.7
Simplification	16	2.3	0	0.0	7	1.1	2	0.6	5	1.1	1	0.3	20	1.8	5	1.8	56	1.3
Patients’ decision	39	5.7	27	5.1	14	2.2	5	1.4	9	1.9	10	3.0	9	0.8	0	0.0	113	2.6
Others **	5	0.7	5	1.0	3	0.5	1	0.3	2	0.4	2	0.6	2	0.2	2	0.7	22	0.5

ATV: atazanavir; BIC: bictegravir; DRV: darunavir; DTG: dolutegravir; EVG: elvitegravir; LPV: lopinavir; RAL: raltegravir; RPV: rilpivirine. * Cardiovascular event (3), malignancy (2), infectious complications (1), multiple events (wasting syndrome, cirrhosis, brain lesions (1)), hospitalization for undefined reasons (3). ** Change of residence (13), physician’s decision for unspecified reason (5), inclusion criteria not fulfilled (2), detention (2).

**Table 3:** Rate ratios (RR) and 95% confidence interval (CI) for any cause treatment interruption in 3656 antiretroviral experienced subjects during the first year of treatment.

	Age-Sex-Adjusted Model				Complete Model *			
Variable	RR	95% CI		*p*-Value	RR	95% CI		*p*-Value
		Low	High			Low	High	
Female sex (ref. Male)	1.22	1.03	1.43	0.02	1.20	1.02	1.43	0.03
Age > 50 years (ref. Age ≤ 50 years)	0.86	0.72	1.02	0.08	1.20	0.99	1.45	0.06
Other ethnicity (ref. Caucasian)	1.17	0.89	1.54	0.26				
Risk factor for HIV acquisition (ref. Heterosexual)								
MSM	0.88	0.68	1.13	0.32	0.95	0.74	1.22	0.70
PWID	1.40	1.16	1.68	0.0004	1.24	0.97	1.58	0.08
Other/unknown	0.99	0.73	1.33	0.94	1.14	0.84	1.53	0.40
HBsAg positive (ref. negative)	0.99	0.70	1.39	0.96				
HCV positive (ref. negative)	1.36	1.16	1.60	0.0001	1.06	0.85	1.32	0.62
CDC stage (ref. A)								
B	1.03	0.84	1.07	0.74	0.93	0.76	1.14	0.50
C	1.50	1.25	1.80	<0.0001	1.21	1.00	1.47	0.046
Baseline detectable HIVRNA (ref. undetectable)	1.83	1.55	2.15	<0.0001	1.36	1.11	1.66	0.003
Baseline CD4+ (ref. < 250), cells/mm^3^								
CD4+ 250–500	0.85	0.69	1.04	0.11	0.74	0.60	0.90	0.002
CD4+ ≥ 500	1.58	1.31	1.92	<0.0001	0.79	0.63	0.98	0.04
Cohort (ref. DTG)								
LPV	2.72	2.08	3.55	<0.0001	1.92	1.41	2.60	<0.0001
ATV	2.57	1.96	3.37	<0.0001	2.03	1.51	2.74	<0.0001
DRV	1.30	0.96	1.77	0.09	1.06	0.77	1.46	0.73
RPV	1.70	1.18	2.44	0.004	1.85	1.29	2.66	0.0008
RAL	0.97	0.67	1.50	0.88	0.79	0.54	1.16	0.23
EVG	1.91	1.35	2.71	0.0003	1.87	1.32	2.65	0.0004
BIC	0.81	0.50	1.32	0.40	0.88	0.54	1.44	0.61

ATV: atazanavir; BIC: bictegravir; CI: confidence interval; CDC center for disease control; DRV: darunavir; DTG: dolutegravir; EVG: elvitegravir; Hepatitis B surface antigen; HCV; hepatitis C virus; LPV: lopinavir; MSM: men who have sex with men; PWID: people who inject drugs; RAL: raltegravir; RPV: rilpivirine; RR: rate ratio. * unconditional multiple logistic regression: the complete model equation included all variables significantly associated with the outcomes in the age- and sex-adjusted analyses.

**Table 4:** Rate ratios (RR) and 95% confidence interval (CI) for any cause treatment interruption in 749 antiretroviral naive subjects during the first year of treatment.

	Age-Sex-Adjusted Model				Complete Model *			
Variable	RR	95% CI		*p*-Value	RR	95% CI		*p*-Value
		Low	High			Low	High	
Female sex (ref. Male)	1.01	0.66	1.53	0.96	1.03	0.68	1.55	0.89
Age > 50 years (ref. Age ≤ 50 years)	1.04	0.69	1.58	0.84	1.04	0.69	1.58	0.84
Other ethnicity (ref. Caucasian)	1.00	0.60	1.68	0.99				
Risk factor for HIV acquisition (ref. Heterosexual)								
MSM	0.73	0.48	1.12	0.15				
PWID	1.11	0.62	1.96	0.73				
Other/unknown	1.25	0.74	2.10	0.40				
HBsAg positive (ref. negative)	0.64	0.25	1.66	0.36				
HCV positive (ref. negative)	1.53	0.97	2.41	0.06				
CDC stage (ref. A)								
B	0.86	0.53	1.40	0.52	0.75	0.47	1.20	0.23
C	1.58	1.09	2.29	0.02	1.11	0.75	1.63	0.60
Baseline CD4+ (ref. < 250), cells/mm^3^								
CD4+ 250–500	0.69	0.47	1.02	0.06				
CD4+ >= 500	0.67	0.42	1.06	0.09				
Cohort (ref. DTG)								
LPV	1.87	1.28	2.72	0.001	1.80	1.22	2.67	0.003
ATV	0.70	0.18	2.79	0.61	0.73	0.19	2.78	0.64
DRV	0.94	0.45	1.95	0.86	0.92	0.44	1.94	0.83
RPV	0.24	0.09	0.67	0.006	0.24	0.09	0.66	0.006
RAL	1.40	0.65	2.97	0.39	1.34	0.63	2.85	0.45
EVG	0.57	0.29	1.12	0.10	0.56	0.29	1.11	0.10
BIC	0.87	0.37	2.05	0.75	0.84	0.35	1.99	0.69

ATV: atazanavir; BIC: bictegravir; CI: confidence interval; DRV: darunavir; DTG: dolutegravir; EVG: elvitegravir; LPV: lopinavir; MSM: men who have sex with men; PWID: people who inject drugs; RAL: raltegravir; RPV: rilpivirine. * unconditional multiple logistic regression: the complete model equation included all variables significantly associated with the outcomes in the age- and sex-adjusted analyses.

**Table 5:** Hazard ratios (HR) and 95% confidence interval (CI) for any cause treatment interruption in 3656 antiretroviral experienced subjects during the entire follow-up.

	Age-Sex-Adjusted Model				Complete Model *			
Variable	HR	95% CI		*p*-Value	HR	95% CI		*p*-Value
		Low	High			Low	High	
Female sex (ref. Male)	1.10	0.98	1.23	0.12	1.15	1.01	1.31	0.04
Age > 50 years (ref. Age ≤ 50 years)	0.72	0.63	0.81	<0.0001	1.12	0.97	1.28	0.13
Other ethnicity (ref. Caucasian)	1.28	1.05	1.57	0.01	1.44	1.17	1.77	0.0005
Risk factor for HIV acquisition (ref. Heterosexual)								
MSM	1.00	0.86	1.19	0.91	1.12	0.95	1.32	0.19
PWID	1.36	1.19	1.55	<0.0001	1.21	1.01	1.45	0.04
Other/unknown	0.97	0.78	1.19	0.75	1.19	0.96	1.46	0.11
HBsAg positive (ref. negative)	1.18	0.95	1.47	0.12				
HCV positive (ref. negative)	1.31	1.17	1.46	<0.0001	1.06	0.90	1.24	0.48
CDC stage (ref. A)								
B	1.00	0.88	1.15	0.94	0.93	0.81	1.07	0.29
C	1.36	1.20	1.56	<0.0001	1.13	0.98	1.29	0.08
Baseline detectable HIVRNA (ref. undetectable)	1.86	1.66	2.09	<0.0001	1.25	1.09	1.44	0.002
Baseline CD4+ (ref. < 250), cells/mm^3^								
CD4+ 250–500	0.67	0.59	0.76	<0.0001	0.79	0.69	0.90	0.0006
CD4+ >= 500	0.53	0.46	0.60	<0.0001	0.82	0.70	0.96	0.01
Cohort (ref. DTG)								
LPV	4.37	3.66	5.21	<0.0001	3.48	2.84	4.27	<0.0001
ATV	2.61	2.15	3.17	<0.0001	2.30	1.86	2.84	<0.0001
DRV	1.32	1.08	1.61	0.007	1.16	0.93	1.44	0.19
RPV	1.62	1.20	2.19	0.002	1.76	1.30	2.37	0.0002
RAL	1.16	0.93	1.46	0.18	1.05	0.83	1.32	0.69
EVG	2.07	1.62	2.64	<0.0001	2.12	1.66	2.72	<0.0001
BIC	0.86	0.57	1.29	0.46	0.90	0.60	1.36	0.62

ATV: atazanavir; BIC: bictegravir; CI: confidence interval; CDC center for disease control; DRV: darunavir; DTG: dolutegravir; EVG: elvitegravir; Hepatitis B surface antigen; HCV; hepatitis C virus; HR: hazard ratio; LPV: lopinavir; MSM: men who have sex with men; PWID: people who inject drugs; RAL: raltegravir; RPV: rilpivirine. * Cox proportional hazard regression model: the complete equation included all variables significantly associated with the outcomes in the age- and sex-adjusted analyses.

**Table 6:** Hazard ratios (HR) and 95% confidence interval (95% CI) for any cause treatment interruption in 749 antiretroviral naive subjects during the entire follow-up.

	Age-Sex-Adjusted Model				Complete Model *			
Variable	HR	95% CI		*p*-Value	HR	95% CI		*p*-Value
		Low	High			Low	High	
Female sex (ref. Male)	1.11	0.83	1.48	0.49	1.05	0.76	1.46	0.76
Age > 50 years (ref. Age ≤ 50 years)	1.18	0.88	1.58	0.26	1.15	0.86	1.56	0.35
Other ethnicity (ref. Caucasian)	1.28	0.90	1.83	0.17				
Risk factor for HIV acquisition (ref. Heterosexual)								
MSM	0.73	0.54	0.98	0.04	0.89	0.65	1.22	0.48
PWID	1.38	0.95	2.01	0.09	0.83	0.52	1.33	0.43
Other/unknown	1.03	0.69	1.55	0.88	1.09	0.72	1.64	0.69
HBsAg positive (ref. negative)	0.90	0.54	1.52	0.70				
HCV positive (ref. negative)	1.87	1.37	2.56	<0.0001	1.58	1.05	2.39	0.03
CDC stage (ref. A)								
B	1.08	0.80	1.46	0.60	0.85	0.62	1.17	0.31
C	1.81	1.38	2.39	<0.0001	1.12	0.80	1.57	0.50
Baseline CD4+ (ref. <250), cells/mm^3^								
CD4+ 250–500	0.51	0.38	0.67	<0.0001	0.80	0.58	1.13	0.21
CD4+ >= 500	0.61	0.45	0.83	0.001	1.01	0.69	1.46	0.97
Cohort (ref. DTG)								
LPV	3.12	2.35	4.16	<0.0001	2.74	1.97	3.81	<0.0001
ATV	1.83	0.91	3.68	0.09	1.64	0.79	3.38	0.18
DRV	1.13	0.63	2.02	0.68	1.10	0.60	2.00	0.76
RPV	0.83	0.17	0.66	0.002	0.36	0.18	0.72	0.004
RAL	1.89	1.01	3.54	0.047	1.89	1.01	3.55	0.048
EVG	1.04	0.70	1.54	0.85	1.10	0.74	1.64	0.65
BIC	1.51	0.80	2.85	0.20	1.54	0.81	2.91	0.19

ATV: atazanavir; BIC: bictegravir; CI: confidence interval; CDC center for disease control; DRV: darunavir; DTG: dolutegravir; EVG: elvitegravir; Hepatitis B surface antigen; HCV; hepatitis C virus; HR: hazard ratio; LPV: lopinavir; MSM: men who have sex with men; PWID: people who inject drugs; RAL: raltegravir; RPV: rilpivirine. * Cox proportional hazard regression model: the complete equation included all variables significantly associated with the outcomes in the age- and sex-adjusted analyses.

## Data Availability

The data that support the findings of this study are available from the corresponding author, upon reasonable request.

## Participating Centers

E. Sarchi, G. Chichino, C. Bolla (Alessandria); A. Saracino, D. Fiordelisi (Bari); L. Calza (Bologna); B. Menzaghi, M. Farinazzo, F. Franzetti (Busto Arsizio); G. Angioni, M. Buffa (Cagliari); P. Maggi, A. Masiello, F. Simeone (Caserta); G. Bruno, B. M. Celesia (Catania); C. Grosso (Cesena); K. Falasca (Chieti); L. Pusterla, G. Chieffo (Como); A. Mastroianni, G. Guadagnino (Cosenza); F. Franzetti, S. Rusconi (Legnano); S. Carradori (Ferrara); F. Vichi, E. Salomoni (Firenze); C.V. Martinelli, F. Lagi (Firenze); A. Di Biagio, C. Dentone, L. Taramasso, M. Bassetti (Genova); E. Pontali, A. Parisini, F. Del Puente (Genova); C. Molteni, A. Pandolfo, S. Piconi (Lecco); S. Rusconi, M. Franzetti (Legnano); G. F. Pellicanò, G. Nunnari (Messina); L. Valsecchi, L. Cordier, S. Parisini, G. Rizzardini (Milano); F. Conti (Milano); A. Bandera, A. Gori (Milano); D. Motta, M. Merli, M. Puoti (Milano); P. Bonfanti, N. Squillace, M. Rossi, E. Pollastri, N. Corti (Monza); S. Martini (Napoli); O. Bargiacchi, P.L. Garavelli (Novara); A. Cascio, M. Trizzino (Palermo); R. Gulminetti, L. Pagnucco (Pavia); G. V. De Socio, G. Gamboni, D. Altobelli, D. Francisci (Perugia); E. Mazzotta, G. Parruti (Pescara); S. Cicalini, R. Bellagamba, A. Antinori (Roma); P. Morelli (Humanitas); G. Cenderello (Sanremo); G. Madeddu, M. S. Mameli (Sassari); G. Orofino, M. Guastavigna (Torino); A. Londero, A. Giacinta, C. Tascini (Udine); G. Battagin, V. Manfrin (Vicenza).
